# Batesian mimicry in the nonrewarding saprophytic orchid *Danxiaorchis yangii*

**DOI:** 10.1002/ece3.7193

**Published:** 2021-03-03

**Authors:** Huolin Luo, Hanwen Xiao, Yuelong Liang, Nannan Liu, Cassidy Turner, Shaolin Tan, Xinghui Chen, Dongjin Xiong, Boyun Yang

**Affiliations:** ^1^ Jiangxi Key Laboratory of Plant Resources School of Life Science Nanchang University Nanchang China; ^2^ Jiangxi Jiulianshan National Nature Reserve Ganzhou China; ^3^ College of Health Solutions Arizona State University Scottsdale AZ USA

**Keywords:** Batesian mimicry, *Danxiaorchis yangii*, deceptive pollination, *Dufourea* spp., *Lysimachia alfredi*

## Abstract

Batesian mimicry, a type of deceptive pollination, is a complicated strategy used by nonrewarding plants to attract pollinators, but some hypotheses concerning this have not been systematically verified. In order to show in detail a case of Batesian mimicry on saprophytic orchid *Danxiaorchis yangii*, the ecological relationship between *Danxiaorchis yangii*, *Lysimachia alfredi* and *Dufourea* spp. was explored. *Lysimachia alfredi* could provide a reward to *Dufourea* sp., whereas *Danxiaorchis yangii* not. The floral morphology and geographical distribution of these two plants were highly overlapping, and the fruit set rate of *Danxiaorchis yangii* was significantly positively correlated with the number of nearby *L. alfredi* individuals. In a glass cylinder experiment, *Danxiaorchis yangii* and *L. alfredi* attracted *Dufourea* spp. through visual signals, but the insect could not distinguish between flowers of the two plants before landing on flowers. The ultraviolet reflection spectra of flowers between the two plant species were highly similar. In the hexagonal color models constructed according to the visual characteristics of bees, the flower color signals of these two plant species highly overlap, indicating that the visual signals of the flowers of the two plants to the pollinator were greatly similar. All of these results provided evidence that *Danxiaorchis yangii* simulated the visual signals of *L. alfredi* through Batesian mimicry, thereby deceptively attracting *Dufourea* spp.

## Graphical abstract

The study provide detailed evidence that *Danxiaorchis yangii* simulates the visual signals of *Lysimachia alfredi* through Batesian mimicry, thereby deceivingly attracting *Dufourea* sp.

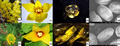

## 1 INTRODUCTION

Approximately one‐third of orchid species provide no reward to their pollinating insects (Shrestha et al., 2020; Trunschke et al., 2019). These orchids have developed many deceptive methods to attract pollinators, such as sexual deception (Rakosy et al., 2017), generalized food deception (Fantinato et al., 2019; Ma et al., 2016), and simulation of rewarding plants (Benadi & Gegear, 2018; Tsuji et al., 2020). Batesian mimicry is a complicated deceptive pollination strategy involving three or more species. The term was originally applied in animal ecology to indicate a nontoxic, edible species that mimics a toxic, inedible species in form, color, and behavior, thereby obtaining safety benefits (Claudel et al., 2019; Sherratt & Peet‐Paré, 2017). Simulation of rewarding plants is equivalent to Batesian mimicry among animals, as both systems involve a signal receiver (pollinator or predator) that mistakes the imitator for a model. Simulation of rewarding plants can therefore be termed Batesian mimicry as well (Schiestl & Cozzolino, 2008). This phenomenon differs, however, between the two types of organisms. In animals, Batesian mimicry functions to repel signal receivers, whereas it functions in plants to attract (Newman et al., 2012; Whitehead et al., 2019).

Batesian mimicry in plant pollination is usually derived from generalized food deception, which mainly attracts insects, such as bees, beetles, small flies, and other generalist pollinators (Bae et al., 2019; Pellissier et al., 2010). Generalized food deception occurs when plants provide false food signals, such as spurs without nectar or fake pollen, to trick insects into foraging on flowers, thereby achieving pollination according to insect foraging behaviors (Nepi et al., 2018, Walsh & Michaels, 2017, Internicola et al., 2008). Most of the species pollinated by bees in Cypripedium belong to this type. Among them, there were as many as 14 species of flower‐visiting insects in *C. plectrochilum*, including bumblebees, small bees, hoverflies, ants and butterflies, but only *Lasioglossum* spp. matches the functional structure of the orchids and is an effective pollinator (Li et al., 2008). Most cases of generalized food deception have no specific mimicry objects, that is, mimicry models. The targets of deception are “naive” insects with no learning experience (Gould & Gould, 1982; Lunau & Wester, 2017). If insects have developed a higher discriminating ability to reduce cheating, however, food‐based mimics change accordingly to match the signals of the models. In such systems of food‐deceptive mimicry, the visual signal is the most important trait for attracting pollinators. Studies to date have shown that the most important feature is color, followed by inflorescence architecture and flower shape, with the least important feature being scent chemistry (Fantinato et al., 2017; Galizia et al., 2005).

Batesian mimicry in plant pollination generally has the following properties: (a) the mimic and model share the same pollinator (Johnson, 2000); (b) geographical distribution and floral morphology overlap among mimic and model (Wüster et al., 2004) (c) the number of mimicked individuals is lower than that of the model (Schiestl, 2005); (d) the mimic and model release similar signals indistinguishable to the pollinator (Jersáková et al., 2016); and (e) the model can act as a “magnetic species,” thereby increasing the mimic's pollinator visitation frequency and fruit set rate. Among these criteria, the fourth and fifth ones have rarely been met in published studies of Batesian mimicry in pollination (Cheney, 2010; de Jager et al., 2016).

Batesian mimicry is the least documented deceptive pollination strategy in orchids and is even controversial in the zoology literature. Few studies have tested most of the above‐mentioned Bates mimicry features, let alone tested all features (Schaefer & Ruxton, 2009, O’Hanlon et al., 2014, Schiestl, 2005). Some early studies only looked for an increase in the reproductive success of a species when the species was growing with its assumed model (Johnson, 1994, Johnson, 2000, Gigord et al., 2002).

*Danxiaorchis yangii*, is a recently described species, and also a fully mycoheterotrophic, extremely endangered orchid with a narrow distribution (Yang et al., 2017). In our early field observations, we found that *Danxiaorchis yangii* might deceptively attract *Dufourea* spp. to pollinate, while *Dufourea* spp. can also pollinate *L. alfredi* and get rewarding. In addition, the two plant species bloomed at the same time and were distributed together. Therefore, in this case study, we analyze the potential Batesian mimicry from multiple perspectives such as pollination behavior, geographic distribution of plant species, effect of *L. alfredi* on fruit setting rate of *Danxiaorchis yangii*, and insect visual models. The research results could show a detailed case of Batesian mimicry and provide scientific guidance for resource conservation of *Danxiaorchis yangii*.

## 2 MATERIALS AND METHODS

### 2.1 Study site

This study was carried out in the Jinggangshan National Nature Reserve, Jiangxi Province, China (26°27′–26°40′N, 113°39′–114°23′E; elevation 200–2,120.4 m). The research site, located at the junction of Jiangxi and Hunan provinces, experiences average annual temperature, rainfall, and humidity levels of 14.2℃, 1,866 mm, and 85%, respectively.

### 2.2 Floral morphology investigation

We recorded the floral morphology of *Danxiaorchis yangii* and *L. alfredi*, including flowering duration of a flower, individual, and patch, according to the standard of Dafni (Dafni, 1992). Floral organ morphological data were obtained using a Vernier caliper. On a sunny day, at 8, 10, 12, 14, and 16 o'clock, 30 flowers of *Danxiaorchis yangii* were selected. The flowers were dissected to determine whether there were nectar, oil, related secretion and storage tissues inside. Glucose test paper was used to detect whether there were traces of nectar in the flower.

### 2.3 Investigations of pollinator and pollination behavior

20 individuals of *Danxiaorchis yangii* and *L. alfredi* was selected for pollination observation between 8:00 and 17:00 daily by HD camera (FDR‐AX700, Sony, Japan) and naked eye, to recorded the various flower‐visiting insects and their flower‐visitation behaviors, including behavior before landing, manner of landing, duration on a flower, pollen deposition and removal, and any repeat visits to flowers while carrying pollinia. Pollinating insects were identified by Kunming Animal Institute of the Chinese Academy of Sciences. The species of pollen carried by pollinators were determined by scanning electron microscopy.

Many plants are pollinated at night, so in addition to close pollination observation during the day, simple experiments should be set up to confirm whether pollination occurs at night. If it occurs, close observation of night pollination should be carried out. Because it is more expensive to observe pollination at night. To check for night pollination, 10 individuals of *Danxiaorchis yangii* and *L. alfredi* were randomly selected before flowering. Until the end of the flowering period, the inflorescences were bagged in the daytime and exposed at night. The selected flowers were examined for pollen deposition and removal each day at 8:00.

### 2.4 Breeding system

To prevent insects or alien pollinia from entering flowers prior to study, 280 flowers of *Danxiaorchis yangii* were selected and bagged into seven separate groups over 2 consecutive years (2018–2019). Three to four days before flowering, one of seven unique treatments was applied to each group: (a) continual bagging (bag kept on until flowers faded), (b) emasculation + bagging (pollinium removal followed by bagging); (c) artificial self‐pollination; (d) artificial geitonogamy; (e) artificial xenogamy; (f) gynostemium removal + bagging; and (g) natural (no bagging). The fruit set rate was counted after flowering, and SPSS software was used to analyze differences among pollination methods.

### 2.5 Spatial distribution of *Danxiaorchis yangii* and *L. alfredi*

Ten patches in the distribution range of *Danxiaorchis yangii* were selected, and each circular patch had a radius of 500 m. To analyze the relationship between the natural distribution of *Danxiaorchis yangii* and *L. alfredi*, the geographical coordinates of all individuals of these two species in each patch were recorded and used to generate a scatter plot.

### 2.6 Analysis of the relationship between the fruit set rate of *Danxiaorchis yangii* and the density of *L. alfredi*

Ten 10 × 10 m quadrats, with a *Danxiaorchis yangii* individual at the center, were delineated. For 3 consecutive years (2017–2019), the fruit set rate of *Danxiaorchis yangii* and the number of *L. alfredi* individuals were counted, and their correlation was analyzed.

To further clarify the relationship between the fruit set rate of *Danxiaorchis yangii* and the number of *L. alfredi* individuals, a transplanting test was carried out. A 1 × 1 m quadrat containing a single *Danxiaorchis yangii* individual, which was located at the center, was established. A total of zero, one, two, four, and eight individuals of *L. alfredi* were transplanted into the quadrat, and the transplantation experiment was performed in quadruplicate. The number of pollinators and durations of their visits were recorded during flowering, and the fruit set rate of *Danxiaorchis yangii* was counted after flowering.

### 2.7 Glass cylinder experiment

To investigate whether plants attract pollinators through olfactory or visual signals, an experiment was performed with three types of glass cylinders (Milet‐Pinheiro et al., 2015): (a) a black cylinder with holes, so that odor was emitted without any visual signal (O cylinder); (b) a sealed, transparent cylinder, so that odor was not emitted but visual signal was present (V cylinder); and (c) a transparent cylinder with holes, so that odor was emitted and a visual signal was present (O/V cylinder). A coupled membrane pump (G12/01 EB; Rietschle Thomas, Germany) was used to circulate air through the cylinders (1 L/min), thereby facilitating odor emission from the cylinders with holes. An inflorescence of *Danxiaorchis yangii* or *L. alfredi* was placed in O, V, and O/V cylinders to investigate the mode of attraction for pollinating insects, namely, whether the plant used olfactory or visual signals. Controls were empty O, V, and O/V cylinders. Each of the two species was placed in the three types of cylinders, yielding a total of six unique glass cylinders. Each unique glass cylinder configuration was set up in triplicate and randomly placed in a patch containing *Danxiaorchis yangii* and *L. alfredi*. For 8 consecutive days, the number of visits from pollinating insects was recorded.

### 2.8 Analysis of flower color

10 flowers that just opened from the two plant species were selected for reflectance spectrum detection by following the method used in some classic papers (Dalrymple et al., 2015; Dyer et al., 2012; Shrestha et al., 2019), while the leaf of *L. alfredi* was served as control (Danxiaorchis yangii has no leaf). During operation, reflectance spectra for wavelengths from 300 to 700 nm were recorded using Ocean Optics spectrophotometer (Dunedin, FL, USA) using a PX‐2 pulsed xenon light sources attached with SPECTRASUITE software to PC. We used UV‐reflecting white standard and black standard to calibrate the spectrophotometer. The reflectance of the flower organs that could be seen from the front of the flower were measured, including the sepals, petals, labellums and gynostemium of *Danxiaorchis yangii,* as well as petals, pistils, and stamens of *L. alfredi*. A reflectivity curve based on the raw spectral reflectance data was drawn using OriginPro 6.1 SR1 software.

To represent flower color perception by bees, a hexagon color space model of hymenopteran vision was employed. Our current model was based on an integration range of 300–650 nm and trichromatic photoreceptors with spectral sensitivity peaks at 350 nm (UV), 440 nm (blue: B) and 540 nm (green: G), using a vitamin A1 visual template (Stavenga et al., 1993), which closely matches trichromatic photoreceptors in many bee species (Briscoe & Chittka, 2001). Several previous studies have used this hexagonal model of hymenopteran color vision (Bischoff et al., 2013; Dyer et al., 2012; Ohashi et al., 2015; Shrestha et al., 2014). Hymenopteran color vision is phylogenetically conserved (Briscoe & Chittka, 2001), thus in the absence of receptor sensitivity values of studies species, it is also appropriate to use the general hymenopteran color model (Kemp et al., 2015).

### 2.9 Data analysis

Routine statistical analyses were performed in IBM SPSS (version 19), while linear regression analysis between the fruit setting rate of *Danxiaorchis yangii* and the individual number of *L. alfredi*, as well as analysis of difference in visit frequency, was carried out by GraphPad Prism 8.

## 3 RESULTS

### 3.1 *Dufourea* spp. pollinated *Danxiaorchis yangii* and *L. alfredi*

**FIGURE 1:**
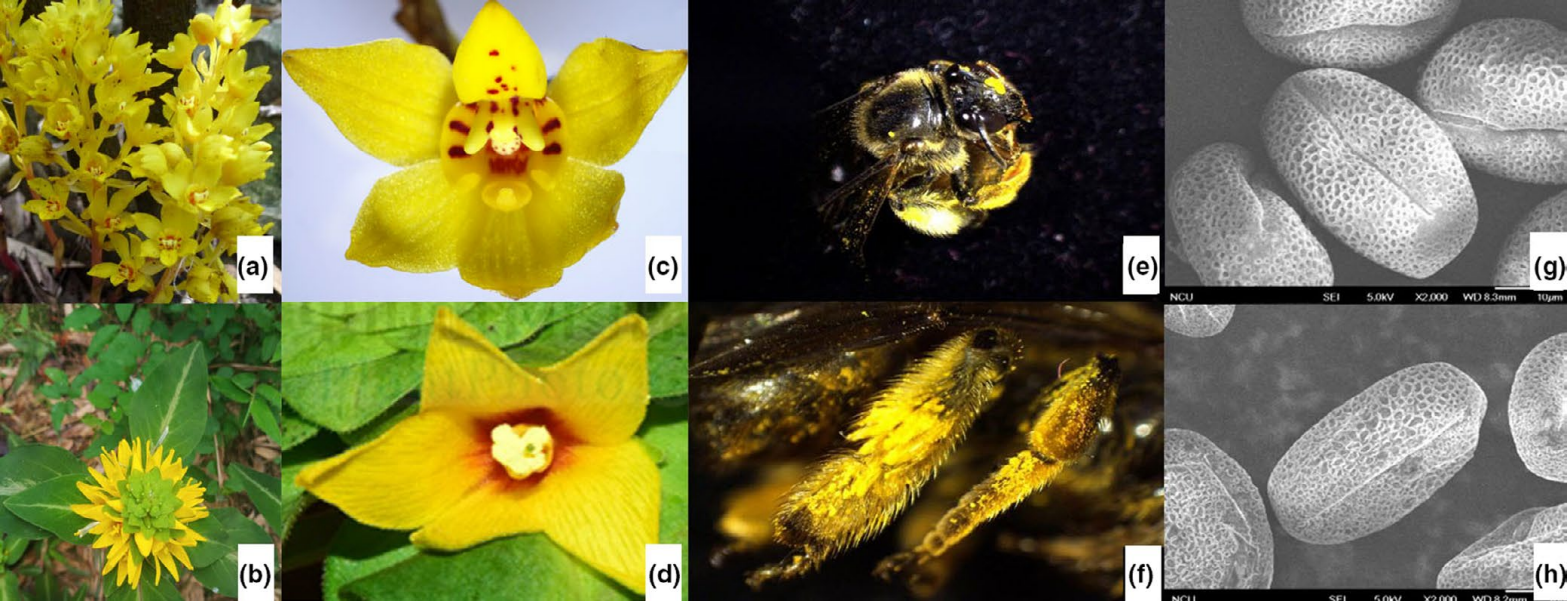
*Dufourea* sp. pollination of *Danxiaorchis yangii* and *Lysimachia alfredi*. (a–b): Inflorescences of *Danxiaorchis yangii* (a) and *L. alfredi* (b); (c–d): Flowers of *Danxiaorchis yangii* (c) and *L. alfredi* (d); (e): Head of a *Dufourea* sp. individual with pollen of *Danxiaorchis yangii*; (f): Electron microscopic image of pollen on the head of a *Dufourea* sp. individual; (g): Appendages of a *Dufourea* sp. individual with pollen of *L. alfredi*; (h): Electron microscopic scan of pollen on the appendages of a *Dufourea* sp. individual

Flowers of *Danxiaorchis yangii* and *L. alfredi* are both yellow, with a purple blotch in the center and no nectar (Figure 1a–d). Neither plant species was pollinated at night. Visiting insects during daylight were captured for scanning of carried pollen via electron microscope. Two types of pollen were carried by *Dufourea* spp., one from *Danxiaorchis yangii* and another from *L. alfredi*. Combined with the insect's flower‐visiting behavior, *Dufourea* spp. was thus determined to be a valid pollinator of the two plant species (Figure 1e–h & Tables S1 and S2). Pollination by *Dufourea* spp. was observed primarily from 11:00–15:00. When *Dufourea* spp. visited *L. alfredi* flowers, it landed directly on the purple blotch, that is, the intersection of petals and stamens. In this location, the insect first touched the anther with its upper jaw, and its three pairs of feet also moved frequently as it attempted to scrape pollen off the stamen, resulting in a large amount of pollen on its thorax and appendages. During this activity, the insect continuously changed its position, which allowed it to cover most of the stamens. After approximately 30–40 s, the insect was covered with pollen and left. When the pollinator visited *Danxiaorchis yangii*, it landed on the labellum and climbed onto the purple blotch located at the junction of the labellum and gynostemium. In this location, the appendage of the labellum is raised, forming a narrow channel with the gynostemium, just enough to accommodate the pollinator's head. The pollinator therefore extended its head into the channel and continued to move around the labellum after exiting. At the same time, the insect also continuously moved its upper jaw and foot and left approximately 2–5 s later. While entering or exiting, an insect could bring in or take out the pollinium of *Danxiaorchis yangii*. The morphological characteristics of *Dufourea* spp., *Danxiaorchis yangii,* and *L. alfredi* were shown in Table 1 and Table S3.

**TABLE 1:** Morphological characteristics of *Dufourea* sp., *Danxiaorchis yangii*, and *Lysimachia alfredi*

Species	Items	Number	Mean (mm)
*Dufourea ssp*	Cephalosome breadth	9	3.33 ± 0.14
	Head thickness	9	1.63 ± 0.26
	Pereion breadth	9	3.62 ± 0.40
	thorax thickness	9	3.40 ± 0.21
	Body length	9	11.07 ± 1.36
*Danxiaorchis yangii*	Opening length	20	5.31 ± 0.83
	Opening breadth	20	3.19 ± 0.48
*L. alfredii*	Opening length	20	11.09 ± 0.57
	Opening breadth	20	11.09 ± 0.57

Anatomical observation and glucose test paper showed that no nectar, oil, and related secretion and storage tissues were found in the flowers of *Danxiaorchis yangii*. In addition, as an orchid plant, the pollen of *Danxiaorchis yangii* is clumped and inedible for bees. On the other hand, *L. alfredi* has been reported to provide edible pollen for bees (including *Dufourea* spp.) (Müller, 2018; Simpson & Neff, 1983).

### 3.2 Pollination restrictions caused a low seed set rate

In the breeding system experiment, the fruit set rate under both “emasculation + bagging” and “gynostemium removal + bagging” treatments was zero, which indicated that no apomixis occurs in *Danxiaorchis yangii* and that the sperm–egg combination was necessary for seed formation. Flowers that were bagged during the entire period did not bear fruit, thus indicating the absence of automatic selfing in *Danxiaorchis yangii* and hence implying that seed production must depend on pollinators. The fruit set rate following artificial pollination, including self‐pollination, geitonogamy, and xenogamy, was above 90%. In contrast, the natural fruit set rate was only 23%, which suggested that *Danxiaorchis yangii* had severe pollination restrictions (Table 2).

**TABLE 2:** *Danxiaorchis yangii* fruit set following different breeding system treatments

Treatment	No. of flowers	No. of fruits	Fruit set (%)
Emasculation + bagged	30	0	0
Removed gynostemium + bagged	30	0	0
Artificial self‐pollination	30	27.40 ± 1.81	91.33 ± 6.05
Artificial geitonogamy	30	29.20 ± 0.84	97.33 ± 2.79
Artificial xenogamy	30	28.80 ± 1.64	96.00 ± 5.47
Continued bagging	30	0	0
Natural (unbagged)	60	7.00 ± 1.00	23.33 ± 3.33

### 3.3 The fruit set rate of *Danxiaorchis yangii* was significantly positively correlated with *L. alfredi* inflorescence density

**FIGURE 2:**
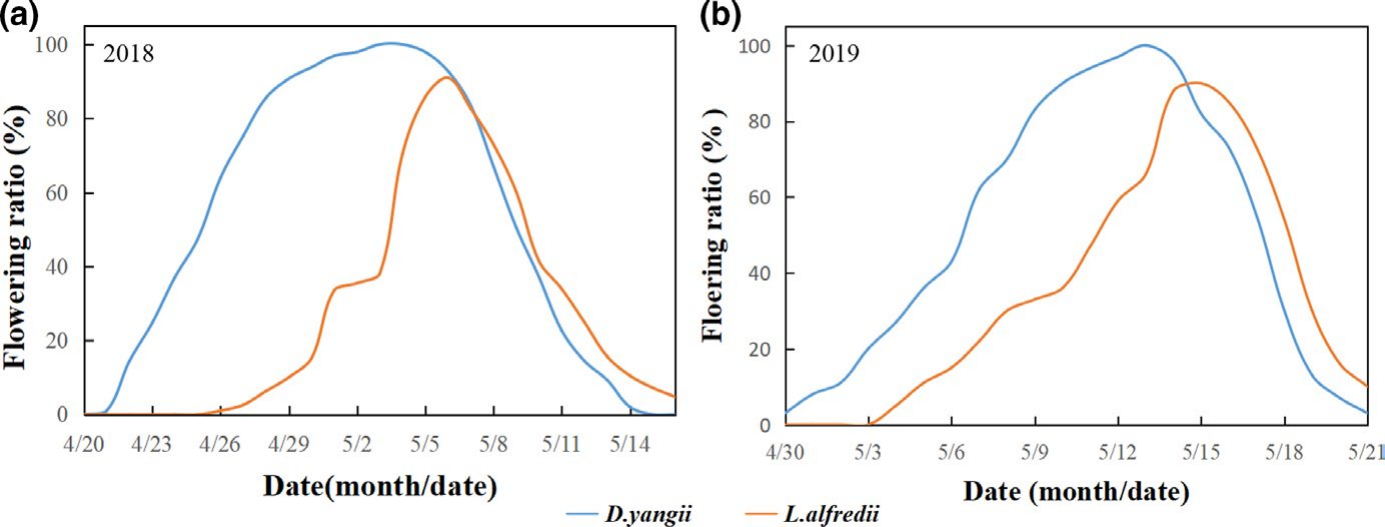
Floral morphology of *Danxiaorchis yangii* and *Lysimachia alfredi*

**FIGURE 3:**
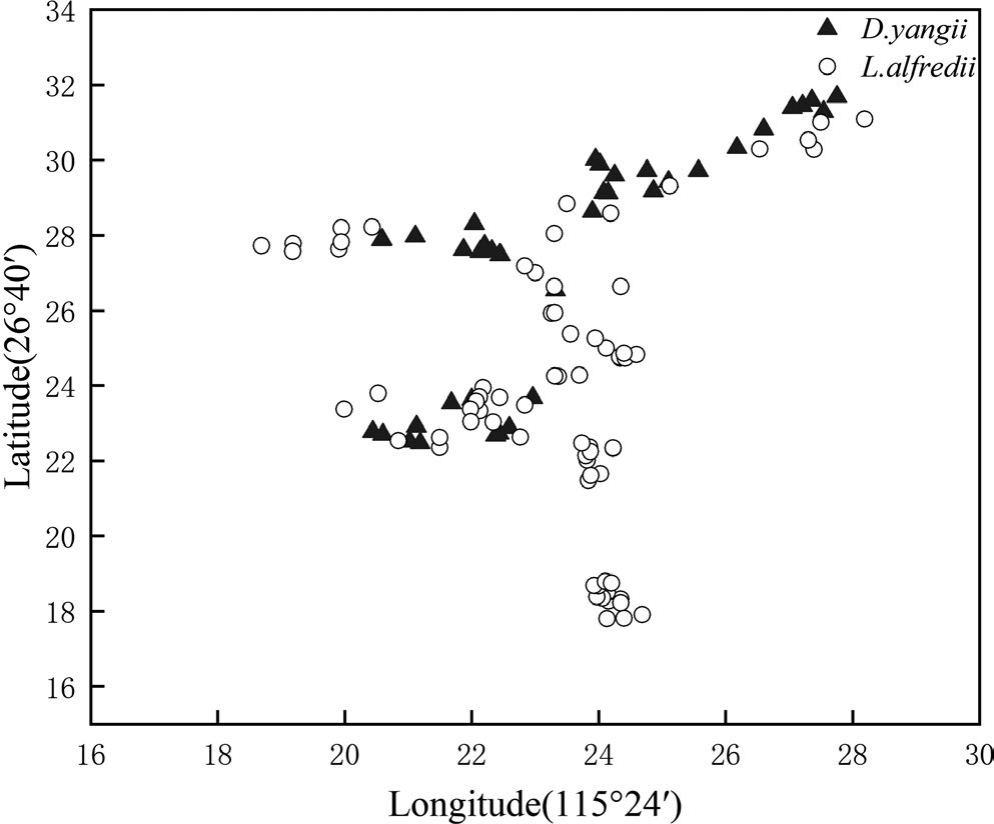
Scatter plot of the geographical distribution of *Danxiaorchis yangii* and *Lysimachia alfredi*

At the study site, flowering of *Danxiaorchis yangii* and *L. alfredi* was highly synchronous, starting in late April, peaking in early May, and ending in mid‐ or late May (Figure 2). The geographical distribution of *Danxiaorchis yangii* at the study site coincided with that of *L. alfredi* (Figure 3). Wherever *Danxiaorchis yangii* was present, *L. alfredi* was found within 50 m, but *Danxiaorchis yangii* occurred at a lower frequency relative to *L. alfredi*.

In the analysis of the relationship between the fruit set rate of *Danxiaorchis yangii* and the density of *L. alfredi*, Pearson correlation coefficients was 0.590, 0.641, and 0.484 in 2017, 2018, and 2019, respectively, and *p*‐values of the two‐tailed test were less than 0.05 (Table 3). It was conclude that a positive correlation existed between the fruit set rate of *Danxiaorchis yangii* and the number of *L. alfredi* individuals.

**TABLE 3:** Correlation between the fruit set rate of *Danxiaorchis yangii* and the number of *Lysimachia alfredi* individuals

		The individual number of *L. alfredii* (2017)	The individual number of *L. alfredii* (2018)	The individual number of *L. alfredii* (2019)
The fruit set of *Danxiaorchis yangii* (2017)	Pearson correlation coefficient	0.59		
	Significance test (2‐tail)	0		
	N	32		
The fruit set of *Danxiaorchis yangii* (2018)	Pearson correlation coefficient		0.484	
	Significance test (2‐tail)		0.05	
	N		20	
The fruit set of *Danxiaorchis yangii* (2019)	Pearson correlation coefficient			0.641
	Significance test (2‐tail)			0.002
	N			20

To complement these analyses based on natural distribution, we performed an experiment involving the transplantation of *L. alfredi* around naturally sited *Danxiaorchis yangii*. In this experiment, the fruit set rate of *Danxiaorchis yangii* increased as the number of surrounding *L. alfredi* individuals increased. When the ratio of *L. alfredi* to *Danxiaorchis yangii* individuals increased to 4:1, however, the fruit set rate of *Danxiaorchis yangii* tended to stabilize or even slightly decrease. A possible reason for this latter result was that *Danxiaorchis yangii* was more readily ignored by pollinators because of the abundance of rewarding *L. alfredi* (Table 4).

**TABLE 4:** The influence of transplanted *Lysimachia alfredi* on *Danxiaorchis yangii*

The individual number of trans‐located *L. alfredii*	0		1		2		4		8	
Year	2018	2019	2018	2019	2018	2019	2018	2019	2018	2019
Number of visits/duration of *Danxiaorchis yangii* only	/	/	8/63	7/46	6/54	3/27	5/31	4/29	3/33	7/65
Number of visits/duration of *L. alfredii* only	/	/	12/95	14/133	14/167	7/209	13/1146	25/1440	104/2942	27/1603
Number of visits/duration between two species	/	/	21/359	8/269	43/784	8/269	53/1580	26/898	23/1372	38/1018
Total visits/duration	19/133	21/361	41/517	29/448	54/1473	29/448	71/2757	55/2367	130/4347	72/2686
The fruit set rate of *Danxiaorchis yangii*	25	37	31	45	47	45.45	57.14	61.54	50	54.54

### 3.4 Both *Danxiaorchis yangii* and *L. alfredi* attracted pollinators through visual signals

According to Tukey's post hoc test, the frequency of visitation to the nine different glass cylinders could be roughly divided into two levels. High levels of visitation were associated with V‐DY (visual signal glass cylinders with *Danxiaorchis yangii*), O/V‐DY (combined signal glass cylinders with *Danxiaorchis yangii*), V‐LA (visual signal glass cylinders with *L. alfredi*), and O/V‐LA, while low levels were found with O‐CK (olfactory signal glass cylinders without plants), V‐CK (visual signal glass cylinders without plants), O/V‐CK, O‐DY, and O‐LA. The visitation frequency in the first four groups was significantly higher than that of the last five groups, with no significant differences detected among the first four groups or last five groups. According to these results, *Danxiaorchis yangii* and *L. alfredi* attracted pollinators by visual signals, and olfactory signals basically did not work. In addition, no significant difference was observed in the frequency of pollinator visits to these two plant species (Table 5).

**TABLE 5:** Frequency of pollinating insects visiting *Danxiaorchis yangii* and *Lysimachia alfredi* based on a glass cylinder experiment

Treatment	Visiting frequency	Treatment	Visiting frequency	Treatment	Visiting frequency
O‐CK	0.00^b^	V‐CK	0.07^b^	O/V‐CK	0.13^b^
O‐DY	0.00^b^	V‐DY	2.63^a^	O/V‐DY	2.43^a^
O‐LA	0.13^b^	V‐LA	3.00^a^	O/V‐LA	2.50^a^

Note

Multiple comparisons were conducted by one‐way ANOVA followed by Tukey's post hoc test. Different lowercase letters indicate significant differences between groups (*p* < .05).

Abbreviations: CK, control; DY, *Danxiaorchis yangii*; LA, *L. alfredi*; O, olfactory signal glass cylinder; O/V, combined signal glass cylinder; V, visual signal glass cylinder.

### 3.5 Flower color models of *Danxiaorchis yangii* and *L. alfredi* were highly similar

**FIGURE 4:**
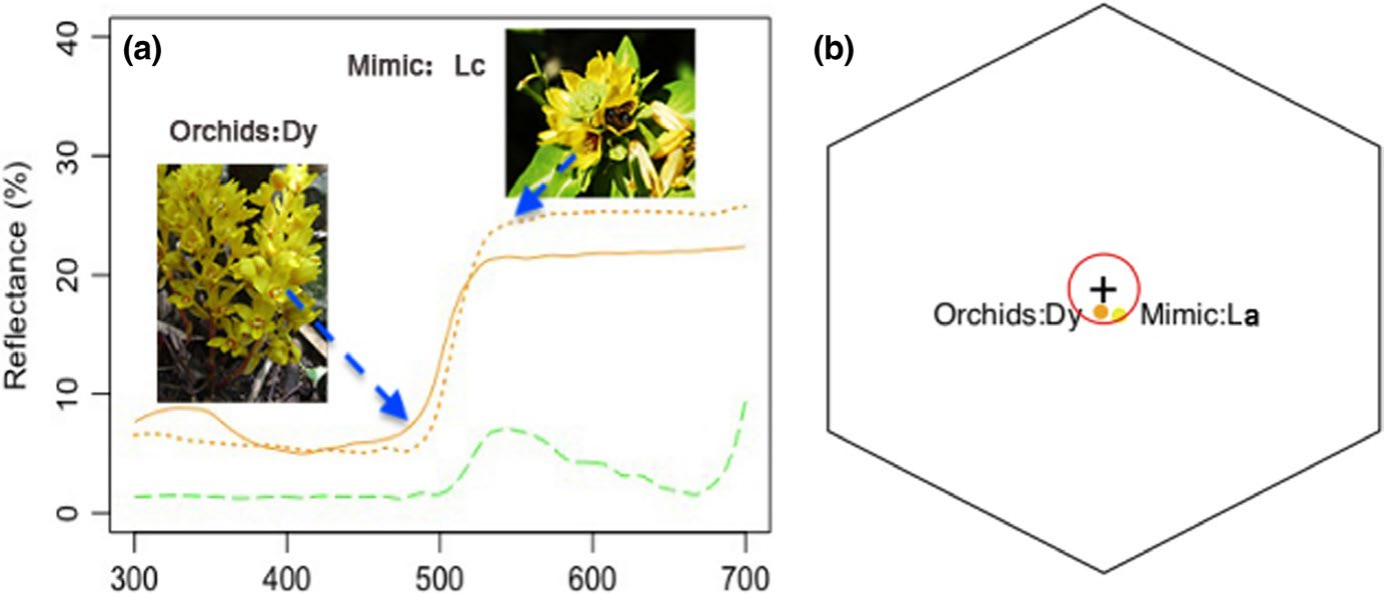
Floral reflectance spectra and hymenopteran color vision model. (a) floral reflectance spectra of two species of flowering plants, orchids (mimic) and non‐orchids (model). Orange solid line = *Danxiaorchis yangii* (Orchidaceae) [Dy], orange dotted line = *L. alfredi* (Primulaceae) [La] and green dashed line = green leaves of *L. alfredi*. (b) Hexagon color model was based on Stavenga et al. (1993) template, red circle shows the 0.11 hexagon unit where insect pollinator cannot discriminate the two species

The spectral reflectance of *Danxiaorchis yangii* and *L. alfredi* flowers was shown in Figure 4a. The spectral reflectance curves of flowers from the two species almost coincided. They remained at a low level until the wavelength reached 500 nm, at which point the reflectance increased and then stabilized between 550 nm and 700 nm. The spectral reflectance of control leaves, however, was different than the flowers of the two species. As shown in the color model in Figure 4b, orchids and model species have 0.07 hex units in hexagon color space, indicating that bee pollinator could not distinguish them.

## 4 DISCUSSIONS

### 4.1 Breeding system and population characteristics

Our breeding system experiment revealed that *Danxiaorchis yangii* could not produce seed without pollination, and no apomixis or self‐compatibility existed. The results indicated that pollinators played a vital role in the successful reproduction of *Danxiaorchis yangii* under natural conditions. The fruit set rate under artificial pollination was much higher than the natural fruit set rate, which indicated a pollination limitation was present in *Danxiaorchis yangii* (Table 2).

*Dufourea* spp. is usually rewarded with nectar or edible pollen when pollinating, but no such substances were found in *Danxiaorchis yangii* flowers. In addition, the shape of *Danxiaorchis yangii* flowers combined with pollinator behavior indicated that the flower could not be served as a breeding spot or shelter for pollinators. Therefore, it was concluded that *Danxiaorchis yangii* mainly attracted *Dufourea* spp. for pollination by deception.

The widely accepted explanation for the deceptive pollination strategy chosen by certain plants is that deceptive pollination could promotes outcrossing and effective pollen output (Jersáková et al., 2006; Kudo, 2006). *Danxiaorchis yangii* has rhizomes and exhibits a scattered distribution pattern in the wild. In a patch, most clumps of *Danxiaorchis yangii* comprise rhizomatous clones. As the genet grows, its flowers generally become gradually surrounded by other flowers from the same individual (Cronberg et al., 2006; Luo et al., 2013). If *Danxiaorchis yangii* can reward pollinators, pollinators will stay in this small area for a long time and travel between flowers. These flowers are likely to be asexual offspring from the same parent, which indirectly reduces the possibility of genetic recombination and impedes plant evolution (Eckert, 2000; Routley et al., 2004). On the other hand, because pollinators could not obtain a reward from *Danxiaorchis yangii*, they left the patch after visiting one to five flowers, thereby reducing the proportion of inbreeding and increasing the chance of outcrossing.

### 4.2 Pollinator specificity

Because approximately 60% of orchid taxa have only one species of pollinator, the family Orchidaceae exhibits a very high degree of pollinator uniqueness (Cozzolino & Widmer, 2005). In some plants with narrow, scattered distributions, inbreeding readily occurs. The evolutionary development of more specialized pollination systems can effectively reduce the risk of inbreeding (Brosi & Briggs, 2013).

In this study, *Dufourea* spp. was the only pollinator of *Danxiaorchis yangii*, and the orchid was found in seven intermittently distributed natural patches, but they were at least several kilometers away from each other. This fragmented distribution largely hinders gene flow between patches. Specific pollinators should significantly improve pollen flow between intermittently distributed patches. In addition, perennial plants with strong asexual reproduction are thought to generally prefer specialized pollination systems, as they can use asexual propagation to overcome the loss of seed yield caused by specialized pollination. The adoption of a specialized pollination system can also reduce a plant's investment in sexual reproduction; this is particularly important for fully mycoheterotrophic orchids, as their nutrient sources are already scarce (Nattero et al., 2010; Shuttleworth & Johnson, 2009).

### 4.3 Mechanism of *Danxiaorchis yangii* pollination

*Lysimachia alfredi* and *Danxiaorchis yangii* shared a pollinator species, the former could provide edible pollen, whereas the latter not. Pollinators therefore stay longer on *L. alfredi* compared with *Danxiaorchis yangii*. The floral morphology and geographical distribution of these two plant species were highly coincident, which raised the question: was there a special relationship between the two plant species?

In a 3‐year quadrat survey, a significant positive correlation was found between the fruit set rate of *Danxiaorchis yangii* and the number of *L. alfredi* inflorescences (Table 3). In addition, a transplanting experiment revealed that in cases where the ratio of *L. alfredi* to *Danxiaorchis yangii* individuals was lower than 4:1 that *L. alfredi* may exert a “magnet species effect” that increases pollinator attraction and visits to *Danxiaorchis yangii* (Table 4) (Molina‐Montenegro et al., 2008).

According to the results of the glass cylinder experiment, *Danxiaorchis yangii* and *L. alfredi* attracted pollinators through visual signals, with the pollinators unable to distinguish between these two plant species. The spectral reflectance of *Danxiaorchis yangii* and *L. alfredi* flowers was highly coincident. In addition, the color model analysis revealed that the flower colors of these two plants were highly similar. No significant differences in floral reflectance spectra have been found between Batesian mimics and their model plants (Kraemer et al., 2015; Peter & Johnson, 2008). The importance of visual signals in food‐deceptive systems has been proven by experiments. Manipulation of visual signal factors, such as UV reflectance, flower color, and flower and inflorescence shapes, has been shown to increase or decrease the frequency of pollinator visits (Newman et al., 2012; Peter & Johnson, 2008, Jersáková et al., 2012).

*Dufourea* spp., a pollen‐feeding insect, has been reported to collect pollen from members of the genus *Lysimachia*. According to our observations, this pollinator behaved similarly on flowers of the two studied plant species. During flower visiting, the target was a purple blotch, and the insect used its upper jaw or foot to explore and collect pollen. Because only one of these two plant species can provide pollen, however, the duration of the pollinator's visit to their flowers was quite different.

Several deceptive orchids, such as *Viola aethnensis* (Ging.) Strobl and *Cephalanthera rubra* (L.) Rich., are assumed to be examples of Batesian mimics, as their fruit set rate is significantly improved when rewarding plants with similar colors are present (Nilsson, 1983, Dafni & Ivri, 1981b, Pellegrino et al., 2008, Dafni & Ivri, 1981a). These cited studies did not determine, however, the relative preferences of pollinators toward those orchids and their putative models; instead, they only demonstrated that having visual signals similar to those of familiar rewarding plants provides more opportunities for the deceptive orchids to be visited. Contrary to one criterion of Batesian mimicry, however, the deceptive orchids are more widely distributed than their putative models. Those putative Batesian mimicry representatives thus attract general pollinators by means of generalized food deception, not Batesian mimicry. In the present study, we have presented the first detailed example of a pollination strategy based on Batesian mimicry in fully mycoheterotrophic orchids, a finding achieved by observations of pollination behavior, glass cylinder experiments, analysis of the relationship between *Danxiaorchis yangii* fruit set rate and the number of *L. alfredi* individuals, and comparative analysis of visual signals from the two plant species.

## 5 CONCLUSIONS

The pollination strategy of *Danxiaorchis yangii* met all criteria of the Batesian mimic hypothesis. First, nonrewarding *Danxiaorchis yangii* and rewarding *L. alfredi* share the same pollinator, and pollination behavior of pollinators on the flowers of these two plant species was similar. Second, the floral morphology of these two plant species substantially overlaps, with *Danxiaorchis yangii* always occurring at a lower frequency relative to *L. alfredi*. Third, the fruit set rate of *Danxiaorchis yangii* increased as the number of surrounding *L. alfredi* individuals increased. Fourth, color models and reflection spectra of flowers of the two plant species are highly similar, while the results of glass cylinder experiments demonstrate that both species attract pollinators through visual signals. Finally, pollinators cannot distinguish between flowers of the two species. We thus concluded that *Danxiaorchis yangii* simulated the visual signals of *L. alfredi* through Batesian mimicry and deceptively attracted *Dufourea* spp. Our results suggested that the deliberate planting of *L. alfredi* next to *Danxiaorchis yangii* communities to attract more *Dufourea* spp. pollinators is a useful strategy for the conservation of *Danxiaorchis yangii* wild resources.

## CONFLICTS OF INTEREST

None declared.

## AUTHOR CONTRIBUTIONS

**Huolin Luo:** Conceptualization (equal); data curation (equal); formal analysis (equal). **Hanwen Xiao:** Conceptualization (equal); data curation (equal); formal analysis (equal). **Yueong Liang:** Investigation (equal); resources (equal). **Nannan Liu:** Formal analysis (supporting). **Cassidy Turner:** Data curation (equal); software (equal). **Shaolin Tan:** Formal analysis (equal). **Xinghui Chen:** Formal analysis (equal). **Dongjin Xiong:** Resources (equal). **Boyun Yang:** Project administration (lead).

## Supporting information

Table S1‐S3

Video S1

Video S2

## Data Availability

Data are available from the Dryad Digital Repository (https://datadryad.org/stash/share/7rzfDefM_AzIV25QCIQu2SapCWhUieEGyuRxD701QR4).
