# Supplementary material for: Batesian mimicry in the nonrewarding saprophytic orchid Danxiaorchis yangii
Source: Ecol Evol. 2021 Mar 3;11(6):2524–34. doi: 10.1002/ece3.7193 (PMC7981215; doi:10.1002/ece3.7193)
Supplement: Supplementary file 1 — Table S1‐S3 [file ECE3-11-2524-s001.doc]

*Table S1 Morphologic characters of Lysimachia alfredii*

|  | Number | Mean（mm） |
| --- | --- | --- |
| Opening length | 20 | 11.09±0.57 |
| Opening breadth | 20 | 11.09±0.57 |
| Petal length | 30 | 22.26±0.77 |
| Petal breadth | 30 | 6.39±0.35 |
| Stigma length | 30 | 9.48±0.27 |
| Stamen length | 30 | 9.36±1.32 |

Table S2 Morphologic characters of *Danxiaorchis yangii*

|  | Number | Mean（mm） |
| --- | --- | --- |
| Opening length | 20 | 5.31±0.83 |
| Opening breadth | 20 | 3.19±0.48 |
| Media sepal length | 20 | 14.72±2.08 |
| Media sepal breadth | 20 | 5.88±0.73 |
| Lateral sepal length | 20 | 13.36±2.03 |
| Lateral sepal breadth | 20 | 6.76±1.00 |
| Petal length | 20 | 13.81±1.79 |
| Petal breadth | 20 | 6.46±1.04 |
| Laballum length | 20 | 9.64±0.84 |
| Laballum breadth | 20 | 4.44±0.57 |
| Column length | 20 | 4.52±0.54 |
| Peduncle length | 20 | 18.09±4.60 |

Table S3 Morphologic characters of *Dufourea* spp*.*

|  | Number | Mean（mm） |
| --- | --- | --- |
| Cephalosome length | 9 | 3.32±0.33 |
| Cephalosome breadth | 9 | 3.33±0.14 |
| Head thickness | 9 | 1.63±0.26 |
| Pereion length | 9 | 4.27±0.23 |
| Pereion breadth | 9 | 3.62±0.40 |
| Chest thickness | 9 | 3.40±0.21 |
| Abdomen length | 9 | 5.74±1.23 |
| Abdomen breadth | 9 | 4.12±0.34 |
| Abdominal thickness | 9 | 2.85±0.27 |
| Body length | 9 | 11.07±1.36 |
